# Elucidation of target implant orientations with the safety range of hip rotation with adduction or abduction during squatting: Simulation based on *in vivo* replaced hip kinematics

**DOI:** 10.3389/fbioe.2022.1023721

**Published:** 2022-11-18

**Authors:** Satoru Harada, Satoshi Hamai, Satoru Ikebe, Daisuke Hara, Hidehiko Higaki, Hirotaka Gondo, Shinya Kawahara, Kyohei Shiomoto, Tetsunari Harada, Yasuharu Nakashima

**Affiliations:** ^1^ Department of Orthopaedic Surgery, Graduate School of Medical Sciences, Kyushu University, Higashi-ku, Fukuoka, Japan; ^2^ Department of Medical-Engineering Collaboration for Healthy Longevity, Kyushu University, Higashi-ku, Fukuoka, Japan; ^3^ Department of Creative Engineering, National Institute of Technology, Kitakyushu College, Kitakyushu, Fukuoka, Japan; ^4^ Department of Artificial Joints and Biomaterials, Faculty of Medical Science, , Kyushu University, Higashi-ku, Fukuoka, Japan; ^5^ Department of Biorobotics, Faculty of Engineering, Kyushu Sangyo University, Higashi-ku, Fukuoka, Japan

**Keywords:** total hip arthroplasty, squatting, kinematics, impingement, anteversion, safe zone

## Abstract

**Objectives:** The study aimed to elucidate target cup orientation and stem anteversions to avoid impingement between the liner and stem neck even at hip rotation with adduction during the deeply flexed posture.

**Methods:** A computer simulation analysis was performed on 32 total hip arthroplasty patients applying patient-specific orientation of the components and *in vivo* hip kinematics obtained from three-dimensional analysis of the squatting motion. The anterior/posterior liner-to-neck distance and impingement were evaluated based on a virtual change in internal/external rotation (0°–60°) and adduction/abduction (0°–20°) at actual maximum flexion/extension during squatting. Cutoff values of cup orientations, stem anteversion, and combined anteversion to avoid liner-to-neck impingements were determined.

**Results:** The anterior liner-to-neck distance decreased as internal rotation or adduction increased, and the posterior liner-to-neck distance decreased as external rotation or adduction increased. Negative correlations were found between anterior/posterior liner-to-neck distances at maximum flexion/extension and internal/external rotation. Anterior/posterior liner-to-neck impingements were observed in 6/18 hips (18/56%) at 45° internal/external rotation with 20° adduction. The range of target cup anteversion, stem anteversion, and combined anteversion to avoid both anterior and posterior liner-to-neck impingements during squatting were 15°–18°, 19°–34°, and 41°–56°, respectively.

**Conclusion:** Simulated hip rotations caused prosthetic impingement during squatting. Surgeons could gain valuable insights into target cup orientations and stem anteversion based on postoperative simulations during the deeply flexed posture.

## Introduction

Total hip arthroplasty (THA) is recognized as an effective surgical treatment for end-stage osteoarthritis (OA) of the hip joint, osteonecrosis of the femoral head (ONFH), and other severe hip diseases to eliminate pain and improve hip function and patient activity with a high level of patient satisfaction (Berry et al., 2002; Ethgen et al., 2004; Mont et al., 2015; Hamai et al., 2016; Hara et al., 2018; Shiomoto et al., 2019). Nevertheless, dislocation after THA remains a major cause of revision despite innovations in prostheses and surgical techniques (Nakashima et al., 2014; Saiz et al., 2019; Shoji et al., 2020; Harada et al., 2021). The revision rate for THA patients with the 32-mm head due to dislocation was 0.60–0.72% at 6-year follow-up (Zijlstra et al., 2017; Hoskins et al., 2022). Even prosthetic impingement between the liner and stem neck (liner-to-neck impingement) is a risk factor for dislocation and accelerated wear and linear fractures, which affect the longevity of implants (Marchetti et al., 2011; Miki et al., 2013).

Squatting is a routine activity in many cultures and requires a greater range of motion of the hip joint (Sugano et al., 2012; Cheatham et al., 2018). Previous reports suggest that *in vivo* squatting kinematics offer no danger of impingement or subsequent dislocation after THA due to sufficient distance between the liner and stem neck (liner-to-neck distance); however, an unintentional internally rotated and adducted posture and lower cup anteversion still remain at risks for posterior dislocation (Harada et al., 2022a). During further analyses in the latter study, simulated unintentional hip rotation was performed without potential disadvantages for dislocation to define impingement-free implant alignment with a safety range of hip rotation. Although there have been several studies on target component orientations simulating impingement using preoperative computed tomography (CT) data (Murphy et al., 2018; Shoji et al., 2020; Vigdorchik et al., 2020), they may not reflect the actual limb position during movement due to a lack of *in vivo* data.

The purpose of the present study, therefore, was to assess the liner-to-neck distance during squatting by changing internal/external rotation and adduction/abduction under a more realistic simulation, which incorporated patient-specific component placements and actual kinematics. In particular, the following question was addressed: What are the target orientations of the components to achieve no liner-to-neck impingement even in an unintentional “dislocation-prone” posture during squatting?

## Materials and methods

### Participants

Between February 2011 and December 2015, a total of 543 patients underwent cementless THA at a single university hospital. Of these, 499 satisfied the following inclusion criteria: 1) alive at the time of the survey, 2) > 1 year since the last surgery, 3) evaluation by a surgeon <1 year, 4) no revision surgery, and 5) no severe dementia or unrelated physical disorder. The survey questionnaire was mailed to all patients, of which 328 patients completed it. The original question investigated the ease of squatting, which was surveyed with four options: 1) yes, “easily possible,” 2) yes, “possible with some support,” 3) no, “impossible,” and 4) no, “have not tried” (Harada et al., 2022a). The subjects were recruited randomly for the study from 211 patients who answered “easily possible” and “possible with some support.” All patients gave informed consent for their data to be included in this institutional review board (IRB number 30–91) approved study. Eligible patients underwent cementless THA as a surgical treatment for severe OA and ONFH between 2011 and 2015 and met the following inclusion criteria: 1) no neuromuscular disorders; 2) no previous surgery of the analyzed hip; 3) no previous surgery or symptoms of other joints or the spine; 4) non-inflammatory arthritis; and 5) use of a 32-mm head during THA. This study consisted of 32 hips in 30 patients including 27 hips in 25 OA patients and 5 hips in 5 ONFH patients (Table 1). There were no cases of dislocation among the patients in this study.

**TABLE 1:** Demographic and radiographic data.

Hips, *n* = 32; patients, *n* = 30	
Age at surgery^a^, y	62.9 ± 9.3 (47–84)
Sex (male; female), hips	14; 18
Body mass index^a^, kg/m^2^	22.8 ± 3.2 (17.7–32.2)
Diagnosis (OA; ONFH), hips	27; 5
Follow-up^a^, y	7.4 ± 1.9 (5.4–8.9)
Preoperative Harris hip score^a^, points	48.5 ± 13.2 (27–81)
Postoperative Harris hip score^a^, points	95.6 ± 3.6 (90–100)
Cup size (48; 50; 52; 54 mm), hips	17; 5; 7; 3
Stem size (#10; 11; 12; 13; 14), hips	6; 3; 12; 9; 2
Prosthetic head diameter (32 mm)	32
Cup inclination^a^, degree	38.1 ± 5.8 (27–48)
Cup anteversion^a^, degree	16.4 ± 6.0 (4–32)
Stem anteversion^a^, degree	33.6 ± 11.4 (7–60)

OA, osteoarthrosis; ONFH, osteonecrosis of the femoral head.

^a^Values are given as the mean ± standard deviations with the range in parentheses.

## Implants

A cementless hemispherical press-fit cup, straight metaphyseal fit stem, and a highly cross-linked ultra-high molecular weight polyethylene liner (AMS and PerFix HA; Aeonian; Kyocera, Kyoto, Japan) were used. The stem-neck angle was 130°. All materials of the femoral heads were alumina ceramic, and all head sizes were 32 mm. The head-neck ratio was 3.56, being the ratio of a 32-mm head to a 9-mm neck diameter.

### Surgical technique

Surgery was performed using a standard posterolateral approach with the lateral decubitus position and combined anteversion (CA) technique (Jolles et al., 2002; Dorr et al., 2009). The femur was prepared first so that femoral anteversion was known before cup placement (Murray, 1993; Dorr et al., 2009; Nakashima et al., 2014). Anteversion of the final broach was measured as the angle between the lower leg’s axis and the trial stem’s axis by flexing the knee and placing the tibia in a vertical position using a manual goniometer. Cup anteversion was then adjusted using a manual manufacturer’s cup inserter with a goniometer, according to the stem anteversion, so that CA ranged from 40° to 60° (Jolles et al., 2002; Nakashima et al., 2014). 45° internal rotation with 20° adduction and 60° internal rotation without adduction are the index positions to check THA posterior stability intraoperatively.

### Orientation of components

Orientation of the acetabular cup and femoral stem was evaluated using postoperative CT (Table 1). Cup inclination was measured as the angle of abduction using the inter-teardrop line as the baseline (radiographic inclination). Cup anteversion was measured as the angle of anteversion in the sagittal plane (radiographic anteversion) (Murray, 1993). Femoral anteversion was measured as the angle of anteversion between the prosthetic femoral neck and the posterior condylar line. The sum of the cup and stem anteversions was used to determine CA (Nakashima et al., 2014).

### Hip kinematics

**FIGURE 1:**
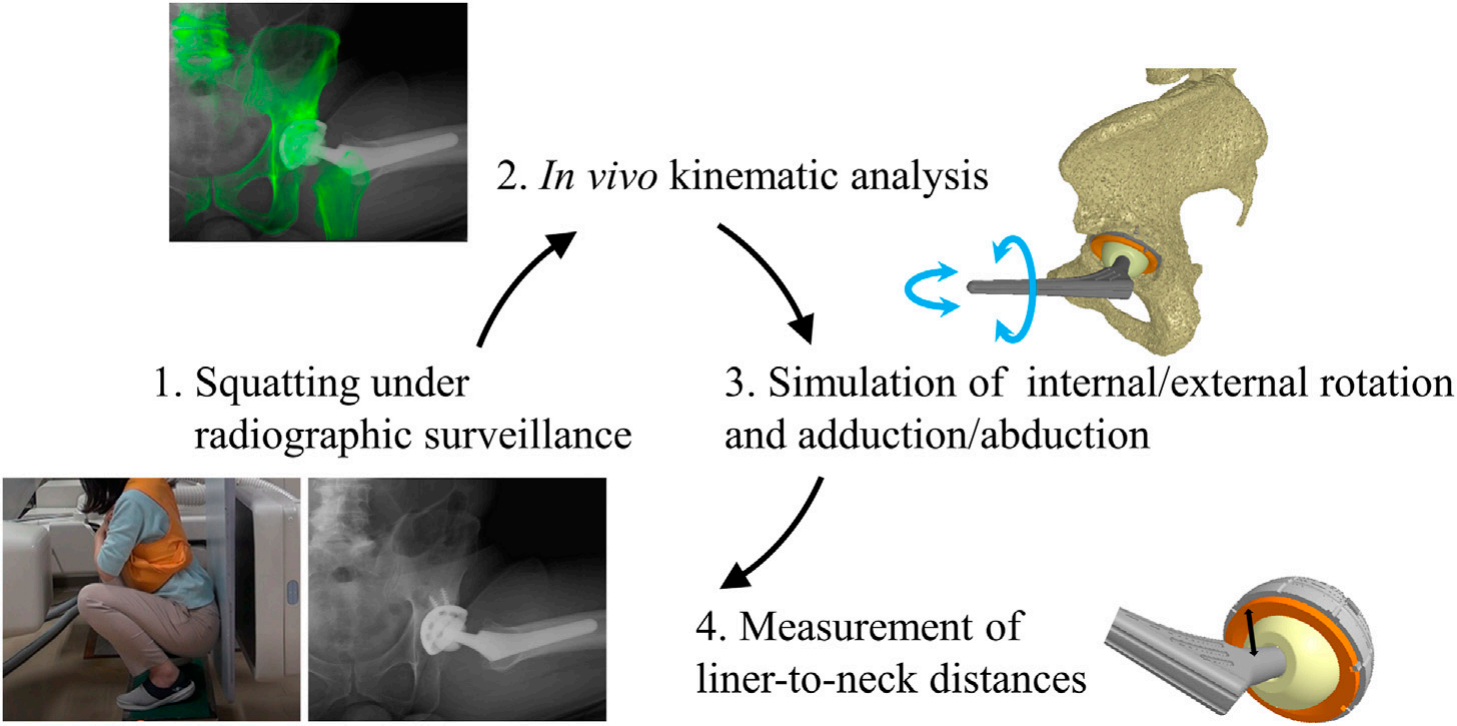
Schema represents the concept of this study. (1) Patients who had undergone total hip arthroplasty stood from a squat position with their heels down, and their hip motions were captured using a flat-panel X-ray detector. (2) The digitally reconstructed radiographs (DRRs) from computed tomography slices were matched to the actual radiographic images to analyze *in vivo* kinematic data [13]. (3) Computer simulations of the hip internal/external rotation and adduction/abduction were performed using *in vivo* implant placement and kinematic data to examine prosthetic impingement at maximum hip flexion and extension during squatting. (4) The minimum distances between the liner and stem neck (liner-to-neck distances) were measured based on three dimensionally (3D) reconstructed images to define an impingement-free implant alignment.

Patients who had undergone THA stood from a squatting position with their heels down under radiographic surveillance, and dynamic hip kinematics were analyzed using density-based, image-matching techniques, as described previously (Figure 1) (Harada et al., 2022a: Hara et al., 2016a; Shiomoto et al., 2019). The squatting position was performed by bending the hip, knee, and ankle joints to descend to the maximum attainable depth. Some of the analysis data have been reported in a previous study (Harada et al., 2022a). Continuous radiographic images during squatting were recorded (Ultimax-i flat-panel X-ray detector [FPD] multipurpose system; Canon, Tochigi, Japan) with a field view of 420 mm × 420 mm, resolution of 0.274 mm × 0.274 mm/pixel, a pulse width of 0.02 s, 80 kV and 360 mA, and a frame rate of 3.5 frames/s. Each patient underwent computed tomography (CT; Aquilion; a 1-mm thickness spanning from the superior edge of the pelvis to Canon) with a 512 × 512 image matrix, a 0.35 × 0.35-pixel dim, and below the knee joint line. This method generated digitally reconstructed radiographs (DRRs) from CT and components’ data, matched the DRRs to the actual radiographs, and calculated the pelvis/acetabular cup and femur/stem orientations (Table 2). The coordinate system of the pelvis was based on the anterior pelvic plane. The center of the acetabular cup was defined as the origin of the coordinate system of the acetabular cup. The coordinate system of the femur was based on the center of the femoral head and the transepicondylar axis, which was defined as the line from the medial to lateral femoral epicondyles (Yoshioka et al., 1987). Hip movement was determined using the Cardan/Euler angle system in the x-y-z order (flexion/extension, adduction/abduction, and internal rotation/external rotation) (Hara et al., 2014). To analyze the orientation of the stem relative to the acetabular cup, local coordinate systems were constructed for each implant to track implant movements. The root mean square errors for bone/implant movement were 0.36/0.43 mm for in-plane translation, 0.37/0.48 mm for out-of-plane translation, and 0.48°/0.52° for rotation (Hara et al., 2014; Hara et al., 2016b).

**TABLE 2:** Hip adduction/abduction and internal/external angles at maximum hip flexion and extension during squatting.

Parameter	Maximum hip flexion	Maximum hip extension
Hip flexion/extension^a^, degree (flexion +, extension −)	80.7 ± 12.3 (60.6–114.2)	1.6 ± 8.4 (−13.0–20.7)
Hip adduction/abduction^a^, degree (abduction +, adduction −)	7.3 ± 5.4 (1.1–17.7)	3.6 ± 3.1 (−4.5–1.4)
Hip internal/external hip rotation^a^, degree (internal +, external −)	−22.7 ± 11.4 (−40.6–2.2)	-10.0 ± 6.5 (−28.7–1.2)

Some of the analysis data have been reported in the previous study (Harada et al., 2022a).

^a^Values are given as the mean ± standard deviations with the range in parentheses.

### Simulation analysis

**FIGURE 2:**
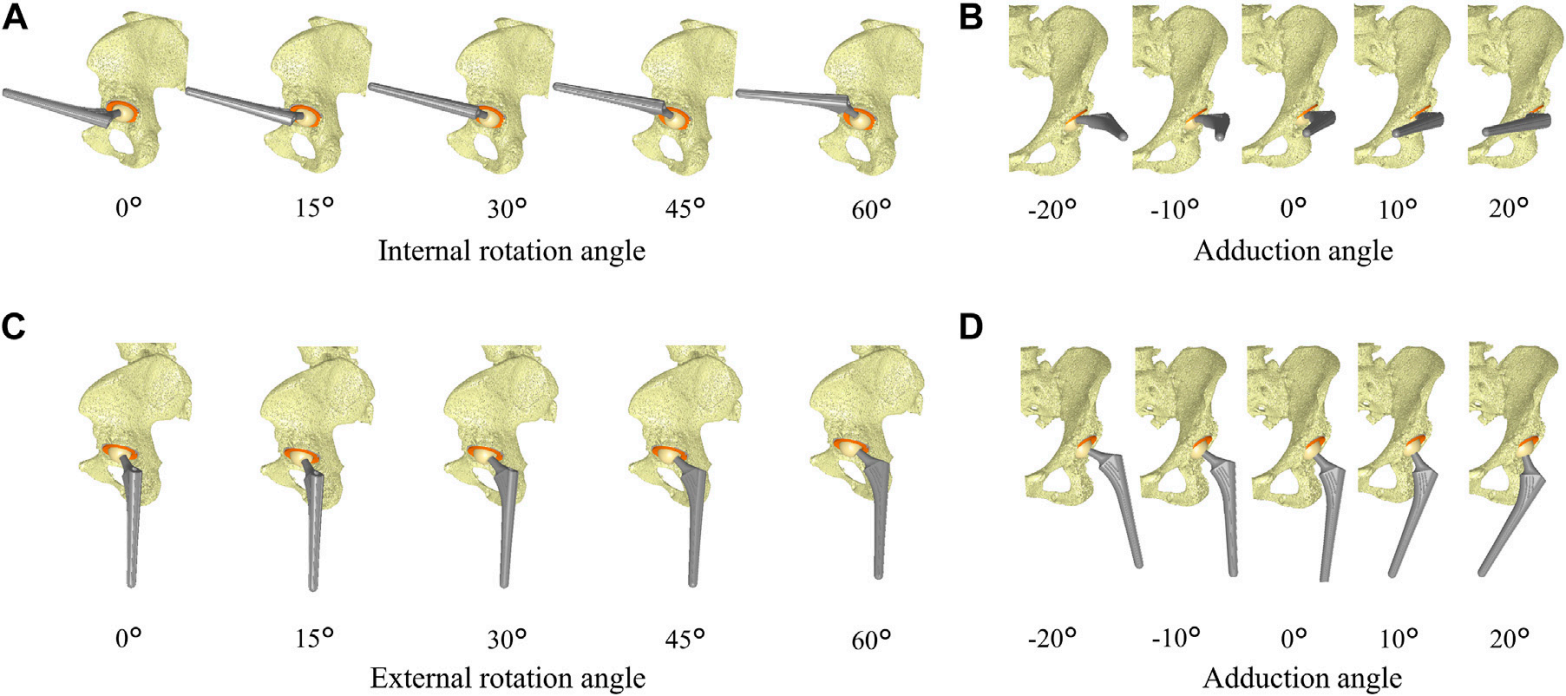
Schema represents the simulation of hip internal/external rotation and abduction/adduction based on *in vivo* implant placement and hip flexion/extension during squatting. The internal rotation and adduction at maximum hip flexion were changed from 0° to 60° in 15° increases **(A)** and from −20° to 20° in 10° increases **(B)**, respectively. The external rotation and adduction at maximum hip extension were changed from 0° to 60° in 15° increases **(C)** and from −20° to 20° in 10° increases **(D)**, respectively.

**FIGURE 3:**
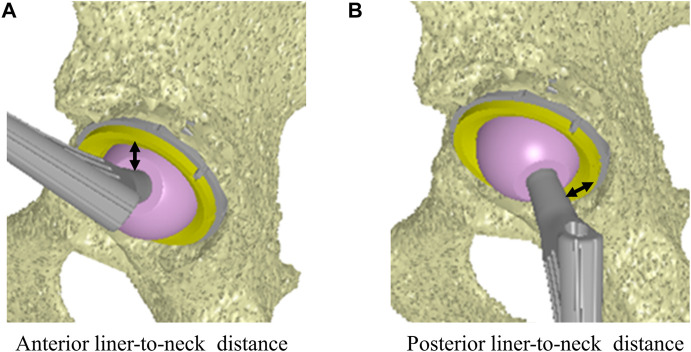
Minimum distance on the anterior side to the stem at the maximum hip flexion as the anterior liner-to-neck distance **(A)**. Minimum distance on the posterior side to the stem at the maximum hip extension as the posterior liner-to-neck distance **(B)**.

Computer simulations were performed using a custom-made software program (Komiyama et al., 2019; Shiomoto et al., 2021) based on *in vivo* data, including patient-specific component placements and kinematics during squatting, to examine liner-to-neck impingement (Figure 1). 1) Internal rotation and adduction/abduction at actual maximum flexion and 2) external rotation and adduction/abduction at actual maximum extension were changed virtually, increasing the angles from 0° to 60° in 15° increases and from −20° to 20° in 10° increases, respectively (Figure 2). The minimum distance between the polyethylene liner and stem neck was measured on the anterior side at maximum hip flexion as the anterior liner-to-neck distance, and the minimum distance on the posterior side at the maximum hip extension as the posterior liner-to-neck distance at each setting was measured using a computer-aided design software program (CATIA V5; Dassault Systèmes, Vélizy-Villacoublay, France) (Hara et al., 2014; Shiomoto et al., 2019; Harada et al., 2022a); the presence or absence of liner-to-neck impingement (Tanino et al., 2008) was also examined (Figure 3). Cup inclination and anteversion, stem anteversion, and CA to avoid anterior impingement at 45° internal rotation with 20° adduction at maximum flexion and posterior impingement at 45° external rotation with 20° adduction at maximum extension, and 60° internal rotation at maximum flexion and posterior impingement at 60° external rotation at maximum extension were examined using receiver operating characteristic (ROC) curves (Shiomoto et al., 2021). All hips were simulated with a flat liner to eliminate the effect of the liner type and the position of the elevated wall.

### Statistical analysis

Statistical analyses were performed by JMP software v.14.0 (SAS Institute, Cary, NC, United States). Correlation between the liner-to-neck distance and internal/external rotation was analyzed using Pearson’s correlation coefficient and linear regression. The cup inclination, cup and stem anteversions, and CA were compared between hips with and without liner-to-neck impingement using Student’s *t*-test and Wilcoxon rank-sum test for normally distributed variables and non-normally distributed variables, respectively. ROC curves were created to calculate the cutoff values of cup inclination, cup and stem anteversions, and CA for anterior/posterior liner-to-neck impingements at 45° internal/external rotation with 20° adduction and at 60° internal/external rotation without adduction. Statistical significance was set as *p* < 0.05. To detect a 14° difference in combined anteversion between liner-to-neck impingement and non-impingement, with a standard deviation of 14°, alpha of 5%, and power of 80%, a sample size of 32 hips was needed in this study (Shiomoto et al., 2021).

## Results

### Liner-to-neck distances and impingements

**FIGURE 4:**
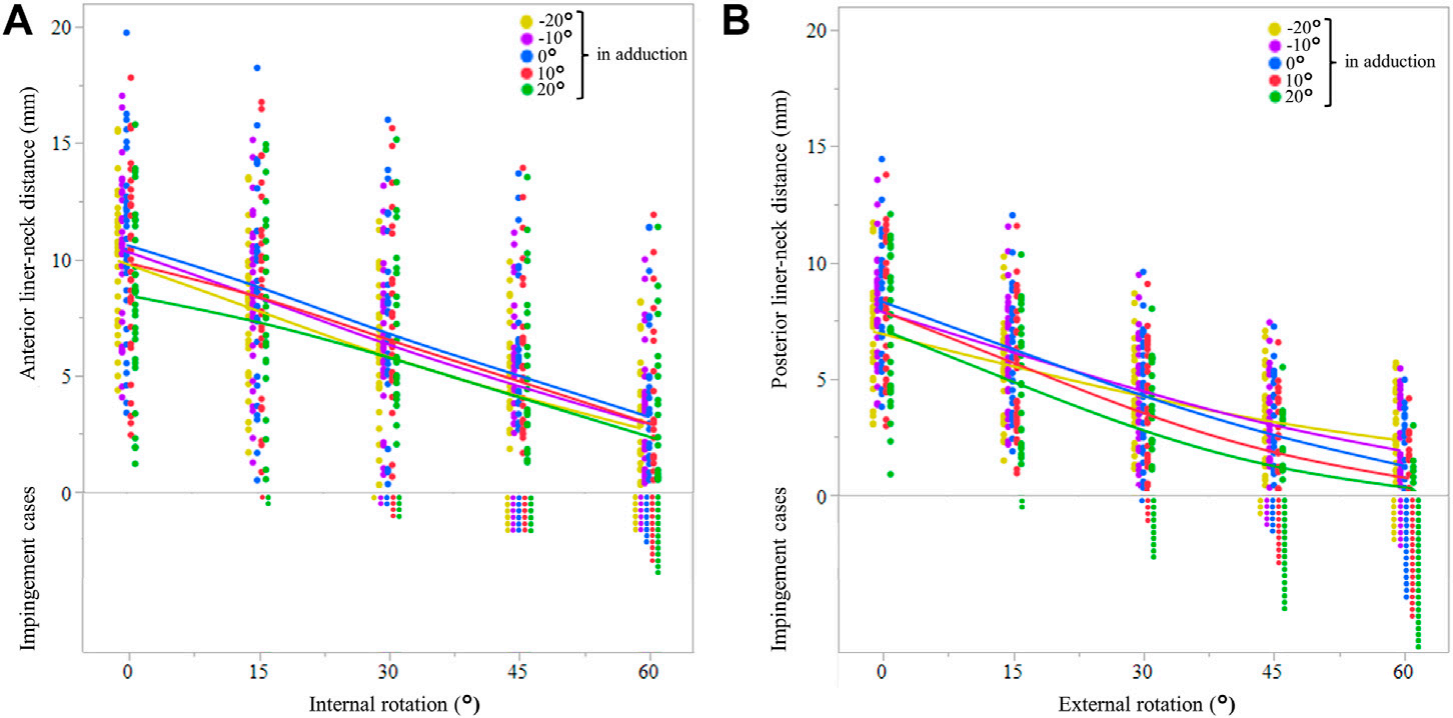
Anterior liner-to-neck distances are based on the simulated internal rotation and adduction at maximum hip flexion **(A)**. Yellow, violet, blue, red, and green solid lines represent average values of −20°, −10°, 0°, 10°, and 20° in adduction, respectively. The dots show the actual values for each patient in each simulation. The posterior liner-to-neck distances are based on the simulated external rotation and adduction at maximum hip extension **(B)**. Yellow, violet, blue, red, and green solid lines represent average values of −20°, −10°, 0°, 10°, and 20° in adduction, respectively. The dots show the actual values for each patient in each simulation.

The anterior liner-to-neck distance decreased as internal rotation or adduction increased, and the posterior liner-to-neck distance decreased as external rotation or adduction increased, respectively. Negative correlations between anterior liner-to-neck distances at maximum flexion and internal rotation were found (*p* < 0.05, Figure 4). The rate of anterior liner-to-neck impingement increased as internal rotation or adduction increased. Anterior liner-to-neck impingements were observed in some cases from 15° internal rotation with 20° adduction/abduction. Similarly, negative correlations between the posterior liner-to-neck distance at maximum extension and external rotation were found (*p* < 0.05, Figure 4). The rate of posterior liner-to-neck impingement at maximum extension increased as external rotation or increased adduction. Posterior liner-to-neck impingements were observed in some cases from 15° external rotation with 20° adduction/abduction. Angles of 10° and 20° adduction significantly decreased both anterior and posterior liner-to-neck distances at 60° rotation compared to 0°, 10°, and 20° abduction (*p* < 0 0.01, Figure 4).

In a 45° internal rotation with 20° adduction, the cup and stem anteversions, and CA with anterior liner-to-neck impingement (*n* = 6 [18%], 15.8 ± 11.3°, 16.7 ± 11.3°, and 32.6 ± 14.0°, respectively) were significantly lower than those without impingement (*n* = 26 [82%], 22.1 ± 8.4°, 37.8 ± 7.7°, and 56.5 ± 14.2°, respectively; *p* < 0 0.05). Meanwhile, in 45° external rotation with 20° adduction, the cup and stem anteversions, and CA with posterior liner-to-neck impingement (*n* = 18 [56%], 23.4 ± 7.7°, 36.7 ± 13.5°, and 60.2 ± 13.2°, respectively) were significantly higher than those without impingement (*n* = 14 [44%], 19.5 ± 10.0°, 29.5 ± 15.7°, and 48.7 ± 18.4°, respectively; *p* < 0 0.05). In a 60° internal rotation with 0° adduction/abduction, the cup and stem anteversions, and CA with anterior liner-to-neck impingement [(*n* = 8 25%, 16.3 ± 10.8°, 16.3 ± 9.8°, and 32.6 ± 12.1°, respectively)] were significantly lower than those without impingement [(*n* = 24 75%, 22.5 ± 8.2°, 35.9 ± 12.8°, and 58.5 ± 12.8°, respectively; *p* < 0 0.05)]. Meanwhile, in a 60° external rotation with 0° adduction/abduction, the cup and stem anteversions, and CA with posterior liner-to-neck impingement [(*n* = 16 50%, 24.5 ± 8.0°, 37.0 ± 14.7°, and 61.6 ± 13.2°, respectively)] were significantly higher than those without impingement [(*n* = 16 50%, 17.3 ± 9.0°, 25.0 ± 12.5°, and 42.4 ± 14.7°, respectively; *p* < 0 0.05)]. Two hips (6%) demonstrated both anterior and posterior liner-to-neck impingements at 60° rotation with 0° adduction/abduction.

### Cutoff values of the cup and stem anteversions and combined anteversion

**FIGURE 5:**
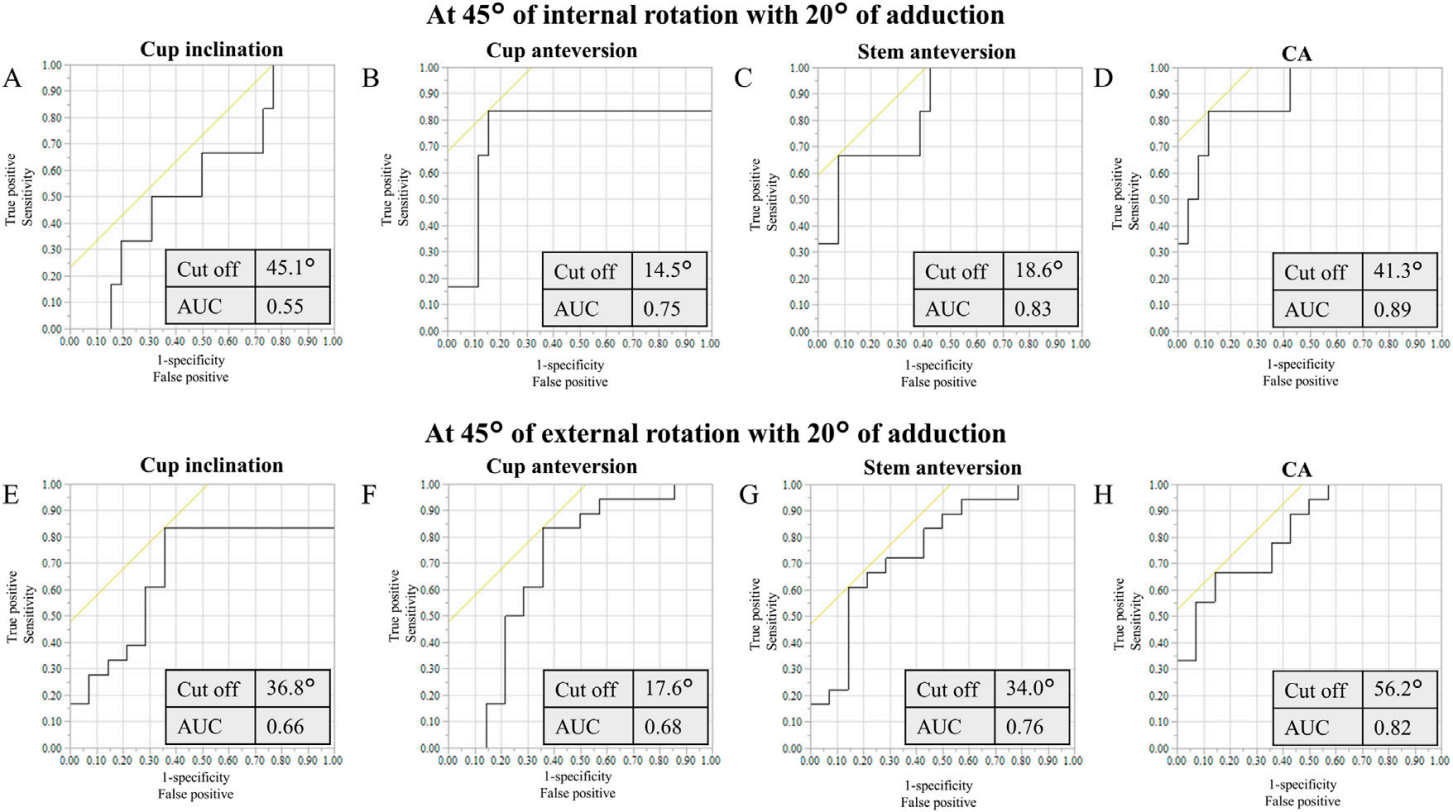
ROC curves of the cup inclination **(A)**, cup anteversion **(B)**, stem anteversion **(C)**, and CA **(D)** to prevent from anterior impingement at maximum hip flexion with simulated 45° internal rotation and 20° adduction. ROC curves of the cup inclination **(E)**, cup anteversion **(F)**, stem anteversion **(G)**, and CA **(H)** to prevent from posterior impingement at maximum hip extension with simulated 45° external rotation and 20° adduction, ROC: receiver operating characteristic, CA: combined anteversion, AUC: area under the curve.

**FIGURE 6:**
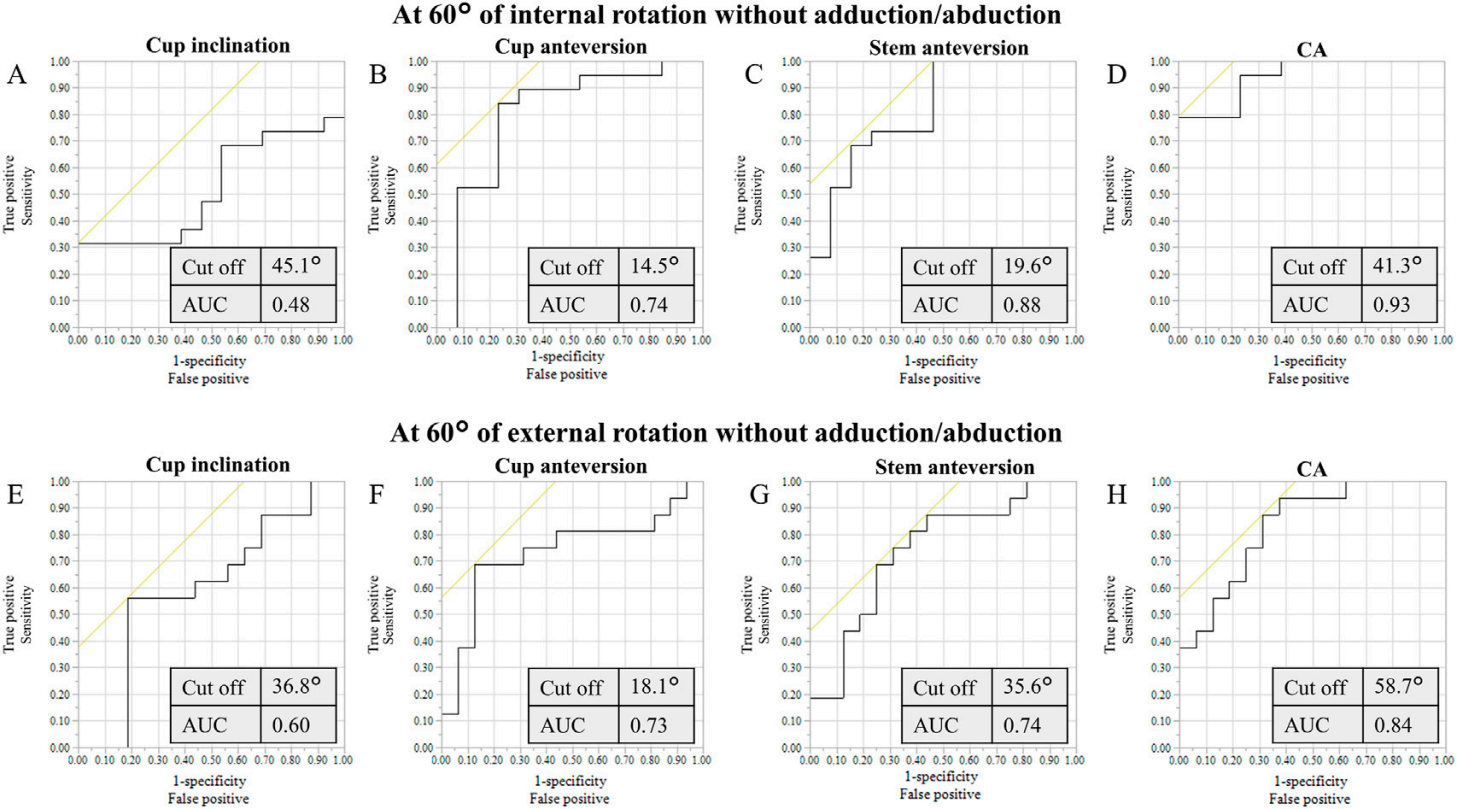
ROC curves of the cup inclination **(A)**, cup anteversion **(B)**, stem anteversion **(C)**, and CA **(D)** to prevent from anterior impingement at maximum hip flexion with simulated 60° internal rotation and 0° adduction. ROC curves of the cup inclination **(E)**, cup anteversion **(F)**, stem anteversion **(G)**, and CA **(H)** to prevent from posterior impingement at maximum hip extension with simulated 60° external rotation and 0° adduction.

Based on ROC curve analyses, the cutoff values of the cup inclination, cup and stem anteversions, and CA for anterior liner-to-neck impingement at 45° internal rotation with 20° adduction were 45.1°, 14.5°, 18.6°, and 41.3°, respectively (Figure 5). Based on ROC curve analyses, the cutoff values of the cup inclination, cup and stem anteversions, and CA for anterior liner-to-neck impingement at 60° internal rotation with 0° adduction were 45.1°, 14.5°, 19.6°, and 41.3°, respectively (Figure 6). The cutoff values of the cup inclination, cup and stem anteversions, and CA for posterior liner-to-neck impingement at 45° external rotation with 20° adduction were 36.8°, 17.6°, 34.0°, and 56.2°, respectively (Figure 5). The cutoff values of the cup inclination, cup and stem anteversions, and CA for posterior liner-to-neck impingement at 60° external rotation with 0° adduction were 36.8°, 18.1°, 35.6°, and 58.7°, respectively (Figure 6). Angles of 60° internal/external rotation with 10° and 20° adduction showed no significant cutoff values to avoid neither anterior nor posterior liner-to-neck impingements.

## Discussion

This is the first study to assess target orientations of components to avoid liner-to-neck impingements based on *in vivo* replaced hip kinematics during squatting. At the actual maximum hip flexion, the anterior liner-to-neck distance significantly decreased as simulated internal rotation or adduction increased. Consequently, anterior liner-to-neck impingements were observed in some cases from 15° or more of internal rotation with 20° adduction. Also, at the actual maximum hip extension, the posterior liner-to-neck distance significantly decreased as simulated external rotation or adduction increased. Consequently, posterior liner-to-neck impingements were observed in some cases from 15° or more of external rotation with 20° adduction. The ranges of the cup and stem anteversions, and CA to achieve neither anterior impingement at maximum hip extension with simulated 45° external rotation and 20° adduction nor posterior impingement at maximum hip flexion with simulated 45° internal rotation and 20° adduction during squatting were 15°–18°, 19°–34°, and 41°–56°, respectively. Using CA could provide a larger range than a separate cup and stem anteversion.

Examining target implant orientations based on postoperative CT data with patient-specific kinematics could mimic the post-THA clinical scenario more closely, guiding the revision of surgical techniques (Harada et al., 2022b). Komiyama et al. reported that a higher vertical center of rotation results in a longer distance between the impingement site and a lower range of flexion and internal rotation (Komiyama et al., 2016). Also, Shoji et al. determined the influence of the stem design on a range of motion (ROM) by using preoperative CT data and simulation software (Shoji et al., 2020). However, previous studies simulating impingement using preoperative CT data in a supine position do not reflect patient-specific *in vivo* kinematics during weight-bearing conditions (Murphy et al., 2018; Shoji et al., 2020; Vigdorchik et al., 2020). This study utilized patients’ specific component position and pelvic tilt during actual deep flexion movements for simulation (Shiomoto et al., 2020; Shiomoto et al., 2021). It has been previously reported that the cutoff values for cup anteversion and combined anteversion to avoid impingement at 60° rotation during chair-rising were 12°–25° and 38°–62°, respectively. However, for kinematics of the hip joint that requires a greater ROM, such as squatting, the target cup position may be more limited to avoid impingement. Squatting is an important function for many daily activities all over the world, as well as a basic movement for strengthening lower limb muscles (Hemmerich et al., 2006; Tang et al., 2014; Cheatham et al., 2018). THA should meet the higher demands of patients and society with regard to functional outcomes (Snijders et al., 2021). Koyanagi et al. (2011) reported that no prosthetic impingement was observed during squatting in an *in vivo* study, suggesting that unexpected postures other than daily activities may lead to dislocation. The maximum hip flexion during squatting was reported to average on 86° (Snijders et al., 2021), which was equivalent to the results of this study: 81°. A previous study also demonstrated that *in vivo* squatting kinematics seem to be safe against impingement and subsequent dislocation (Harada et al., 2022a), but unintentional hip rotation remains a risk. Furthermore, there is widespread interest in improving the impingement-free ROM of the hip after THA. Previous studies have reported that the required ROM for daily activities is 30°–45° of internal/external rotation in hip flexion/extension (Hemmerich et al., 2006; Miki et al., 2007; Nadzadi et al., 2003; Pedersen et al., 2005; Sugano et al., 2012; Yamamura et al., 2007). The target cup anteversion and CA required to avoid impingement at 45° rotation with 20° adduction during squatting were 15°–18° and 41°–56°, respectively, which is significantly lower than that during chair-rising (Shiomoto et al., 2021).

For cup inclination and anteversion, our results were lower than Lewinnek’s safe zone (cup inclination: 40° ± 10°, anteversion: 15° ± 10°) (Lewinnek et al., 1978). Indeed, there have been several reports of dislocations even within Lewinnek’s safe zone (Danoff et al., 2016; Dorr and Callaghan, 2019; Elkins et al., 2015; Tiberi et al., 2015). Widmer and Zurfluh recently reported the target cup anteversion as 20°–28° and CA as 37° (Widmer and Zurfluh, 2004). Hisatome and Doi reported the ideal range of cup anteversion as 15°–35° and the target CA as 42° based on a mathematical formula (Hisatome and Doi, 2011). As previously mentioned, Shiomoto et al. reported that the target cup anteversion and CA were 12°–25° and 38°–62°, respectively (Komiyama et al., 2019; Shiomoto et al., 2021). In the present study, the ranges of cup anteversion and CA to achieve neither anterior nor posterior impingement during squatting were 15°–18° and 41°–56°, respectively. Compared with the reported safe zones, a relatively narrow safety zone was revealed in this study. An optimal implant alignment to prevent prosthetic impingement exists with a sufficient safety margin of hip rotation during squatting. Hips without anterior/posterior liner-to-neck impingement showed significantly higher/lower cup anteversion and CA than those with impingement. These results were comparable with previous studies (Sato et al., 2013; Hara et al., 2014; Harada et al., 2021). As an extreme limb position, the target cup anteversion and CA that achieve no prosthetic impingement at 60° rotation with 10° or 20° adduction did not exist after THA. These data may be beneficial for advising patients after THA regarding postoperative squatting activities in daily life.

Both cup and stem anteversion showed significant relationships with the liner-to-neck distance and postoperative potential ROM (Shiomoto et al., 2021; Harada et al., 2022a). Stem anteversion has been reported to have a strong correlation with preoperative native anteversion when inserting a straight metaphyseal fit stem (Hirata et al., 2014; Park et al., 2015). In hips with excessively low or high amounts of femoral anteversion, changeable neck or cone-type stems may be useful options, by adjusting femoral anteversion to achieve the target CA (Matsushita et al., 2010; Howie et al., 2012). Computer-assisted surgeries such as navigation or robot-assisted systems have been reported as useful tools to verify and achieve the precise orientation of components (Hazratwala et al., 2020; Rhee et al., 2019).

The present study has several limitations. First, only a single component design was analyzed: a hemispherical press-fit cup and a straight metaphyseal fit stem. However, the design was similar to many other components currently available. Second, the simulation was performed with all hips unified to flat liners. Previous studies reported that an elevated liner had a significant effect on posterior impingement, and the results may be different in hips with an elevated liner (Shiomoto et al., 2019). Third, this study excluded a 28-mm head and included only one ball diameter: a 32-mm head (Harada et al., 2022a). Although this study is not directly applicable to THA with a head size larger than 32 mm, our results can still be useful because larger heads increase the impingement-free arc of hip motion (Malik et al., 2009). Fourth, this study did not include hip kinematics in patients with symptomatic lumbar disease. Fifth, we could not find any significant difference (*p* > 0.05) in implant orientations or hip kinematics in a deep squatting position between males and females probably due to a limited number of patients. Further investigation will be necessary to understand a gender-specific safe zone or a patient-specific safe zone in patients with a flat back deformity and/or stiff spine. Sixth, soft tissue has not been considered a limitation to the range of motion before impingement. Seventh, all hip replacements were performed using a standard posterolateral approach. Different approaches could create different postoperative soft tissue environments and hip joint kinematics. Eighth, only one activity rising from a squatting position was considered, and a specific recommendation for target orientations of components in this study could not be adapted to the other activities, such as leg cross, stooping, pivoting, and lunging. Target orientations of components need to be a compromise to satisfy the disparate requirements of different activities (Nadzadi et al., 2003; Pryce et al., 2022).

In conclusion, simulated unintentional hip rotations caused anterior/posterior prosthetic impingement during squatting. The target cup and stem anteversions and CA to avoid prosthetic impingement at 45° internal/external hip rotation with 20° adduction during squatting ranged from 15° to 18°, from 19° to 34°, and from 41° to 56°, respectively. Surgeons could gain valuable insights into target component orientations based on postoperative simulations during the deeply flexed posture.

## Data Availability

The original contributions presented in the study are included in the article/supplementary material; further inquiries can be directed to the corresponding author.
